# Prevalence and determinants of thrombocytopenia in newborn unit at Alexandria University Hospital: a three-year report including 1000 patients

**DOI:** 10.1186/s12887-024-05170-7

**Published:** 2024-12-10

**Authors:** Marwa Mohamed Farag, Mohamed Hazem Goda, Hanan Fawzy Nazir, Ahmed Akram Deghedy

**Affiliations:** Alexandria University Hospital, Pediatric Department, Alexandria, Egypt

**Keywords:** Neonatal thrombocytopenia, Patterns of thrombocytopenia, Mortality prediction in thrombocytopenic neonates

## Abstract

**Background:**

Thrombocytopenia might be the only clinical clue of diseases in neonates. Classification of thrombocytopenia according to severity, onset offset, nadir and duration might help in identification of the etiology.

**Aim:**

This study aims to estimate the prevalence and, identify the determinants and patterns of thrombocytopenia among neonates.

**Method:**

An observational retrospective cohort study was conducted and included records of neonates admitted to neonatal intensive care unit of Alexandria University Maternity Hospital. Data were collected using a checklist and analyzed by SPSS version 20.0. Chi square test and independent sample *t*- test were used to compare different variables between thrombocytopenic and non- thrombocytopenic patients. Logistic regression analysis was carried out in order to identify the independent contribution of various maternal and neonatal variables influencing thrombocytopenia and factors impacting mortality in thrombocytopenic patients.

**Results:**

Four-thousands five hundred neonates, were randomized to have sample of 1011 neonates to be included in the analysis. Patients were divided into thrombocytopenic group (n = 375) and thrombocytopenic group (*n* = 636). Gestational age and birthweight were significantly lower in thrombocytopenic group with *p* values < .001 and .03, respectively. Necrotizing enterocolitis, early onset sepsis and late onset sepsis were the main determinants of neonatal thrombocytopenia with OR (95% CL), 2.25 (1.25–3.98), 4.8 (3.249–7.19) and 6.567(4.083–10.562). severe intraventricular hemorrhage, pulmonary hemorrhage and cardiovascular instability were main predictors of death in thrombocytopenic patients with OR (95% CL), 4 (1.9–8.34), 20.9 (6.7 -64.9), and 5.76 (2.1–15.8), respectively.

**Conclusion:**

Identification of severity and patterns of thrombocytopenia can help the clinician to recognize the etiology and consequently optimize management of thrombocytopenic neonates.

**Supplementary Information:**

The online version contains supplementary material available at 10.1186/s12887-024-05170-7.

## Background

One of the most prevalent hematologic conditions in neonatal intensive care units (NICUs) is thrombocytopenia. It is defined as a platelet count < 150 × 10 ^9^/L in any infant, regardless of gestational age [1]. The prevalence varies according to the demographic population under investigation. Although thrombocytopenia may occur in 1–5% of infants, the prevalence of thrombocytopenia in neonates admitted to NICU is reported at 18–35% of all admissions, and it shows a negative correlation with the gestational age [2]. Several mechanisms are involved in neonatal thrombocytopenia including decrease platelet production, increase platelet destruction and mixed mechanisms [3].

The onset and severity of thrombocytopenia can be used to determine the clinical pattern and aetiology. Thrombocytopenia can develop early, in the first 72 hs of life or late, after the first 72 hs. [1] Early onset neonatal thrombocytopenia is commonly associated with complications related to pregnancy, such as intrauterine growth restriction (IUGR), maternal diabetes, maternal immune thrombocytopenic purpura (ITP), congenital infections or neonatal alloimmune thrombocytopenia [4]. On the other hand, late onset neonatal thrombocytopenia is usually secondary to necrotizing enterocolitis (NEC) or sepsis, and it is usually more severe [5]. The severity of thrombocytopenia can be categorized into: mild (platelet count of 100–150 × 10^3^ /mm^3^), moderate (platelet count of 50- < 50 × 10^3^ / mm^3^) [4]. In most cases, neonatal thrombocytopenia is mild to moderate and resolves without intervention. Severe neonatal thrombocytopenia, however, may result in potentially fatal bleeding, such as pulmonary hemorrhage or intraventricular hemorrhage (IVH), which carries a significant risk of neurodevelopmental impairment [6, 7].

To the best of our knowledge, there is a paucity of epidemiological research on thrombocytopenia that is carried out locally and regionally with a sizable enough sample, thus explaining why such a study is vital. Furthermore, we linked the thrombocytopenia patterns to the etiology in order to help the physician in determining the etiology depending on the clinical patterns.

## Aim of study

This research aimed to assess the prevalence of thrombocytopenia and to determine its contributing factors among newborns at NICU of Alexandria University Maternity Hospital (AUMH). In addition to study patterns of neonatal thrombocytopenia and predictors of mortality among thrombocytopenic neonates.

## Methods

Systematic random sample (*n* = 1011 records) was taken from around four thousand five hundred (total admissions, *n* = 4474 neonates) available records of neonates admitted to NICU of AUMH in the time period from January 1st 2020 to the 31st of December 2022. Of the total admissions, 1011 records were chosen by systematic random sampling pattern, so the chosen sample was representative for the whole records. If the record chosen has incomplete data, it was discarded and another one was taken (*n* = 15 patients’ record). The study was approved by the ethics committee of the Alexandria University with, ID number 0107515, IRB number 00012098, and FWA number 00018699.

The prevalence of thrombocytopenia among admitted patients was calculated. The demographic characteristics of patients with thrombocytopenia versus those without it were evaluated. In addition, the clinical and CBC-presentations of patients with thrombocytopenia were compared to non-thrombocytopenic patients. The degree of thrombocytopenia, maternal and neonatal risk factors, potential causes, time of onset, nadir, duration of different causes of thrombocytopenia, and predictors of thrombocytopenia were studied. The outcomes of thrombocytopenic patients and the tools of management were also examined. All data used in the current work were collected from patients’ files and printed laboratory results in patients’ files.

Sample size calculation and randomization: A minimal sample size of 800 records was needed to estimate the prevalence of thrombocytopenia, with a precision 2% (assuming a prevalence of thrombocytopenia among neonate at 24%to 26%, according to previous studies [8, 9], with 95% confidence level. Software: NCSS 2004 and PASS 2000 software. Systematic random sample from available records was obtained from 4474 available records in the time period from January 1st 2020 to the 31st of December 2022. 1011 records were obtained by systematic random sampling pattern so that the chosen sample were representative for the whole records. If the record chosen was of incomplete data, then, it was discarded, and another one was taken, instead. The person who did the randomization was not involved in the study.

Statistical calculation: Data were fed to the computer and analyzed using IBM SPSS software package version 20.0. The Kolmogorov–Smirnov test was used to verify the normality of distribution. Qualitative data were described using number and percent. Quantitative data were described using range (minimum and maximum), mean, and standard deviation, median and interquartile range (IQR). Significance of the obtained results was judged at the 5% level. Student t-test, Monte Carlo test, Chi-square test, and Fisher Exact test were used for comparison between the two groups regarding different variables. Multiple logistic regression analysis was performed to detect the independent contribution of different maternal and neonatal factors affecting thrombocytopenia and factors affecting mortality in thrombocytopenic patients. Enter method was selected and Nagelkerke R Square was used to detect the amount of variance accounted by predictors include in the model.

## Results

Alexandria University Maternity Hospital (AUMH) is a tertiary center that receives critical deliveries from North Egypt. Thrombocytopenia was seen in 37.1% of the 1011 patients hospitalized to the NICU of Alexandria University Hospital, according to our study. In our study we describe a mean age of onset of thrombocytopenia (4.49 ± 9.17 days) ranged from (1to 76 days).

Of total 375 thrombocytopenic patients, 171 (45.6%) patients are asymptomatic, 118 (31.47%) patients had purpura/petechiae, 86 (22.93%), had ecchymosis. Bleeding tendency at time of thrombocytopenia are 150/375 (40.3%) patients. Bleeding manifestations and pulmonary hemorrhage were significantly higher in thrombocytopenic group, Table 1. At time of thrombocytopenia, 132/375 (38.3%) patients had IVH and 118/375 (35.4%) patients had patent ductus arteriosus (PDA). BPD was present in 31 of 375 thrombocytopenic patients and ROP was present in 15 of 375 thrombocytopenic patients. PDA, bronchopulmonary dysplasia (BPD) and retinopathy of prematurity (ROP), and IVH were significantly higher in thrombocytopenic group, Table 1. Moreover, PDA, IVH, intrauterine growth retardation (IUGR), sepsis and mortality were significantly related to severe thrombocytopenia, S-Table 1.

**Table 1:** Comparison between thrombocytopenic group vs nonthrombocytopenic group regarding sociodemographic and perinatal data

	***Study groups***				**Test of significance (p)**
	**Thrombocytopenic patients (No. = 375)**		**Non-Thrombocytopenic patients (No. = 636)**		
	***No***	***%***	***No***	***%***	
**Sex**					**(X**^**2**^** = 2.9, *P* = .08)**
• **Male**	195	52.00	366	57.6	
• **Female**	180	48.00	270	42.4	
**IUGR**					**(X**^**2**^ = 4.7, *P* = .03*)
• **Yes**	37	9.9	39	6.1	
• **No**	338	90.1	597	93.9	
**Mode of delivery**					**(X**^**2**^** = 3.5, *P* = .06)**
• **NVD**	131	34.93	186	029.25	
• **CS**	244	65.07	450	70.75	
**Maternal thrombocytopenia (No = 601)**					**(X**^**2**^ = 4.39, *P* = .04*)
• **Yes**	19	8.2	14	3.8	
• **No**	214	91.8	354	96.2	
**Family history of thrombocytopenia (No = 659)**					**(X**^**2**^ = 4.9, *P* = .03*)
• **Yes**	24	8.5	15	4	
• **No**	260	91.5	360	96	
**Gestational age(weeks)**					
**Mean ± SD**	**33.8 ± 3.9**		**35.1 ± 3.5**		(t = 4.7, *P* < .001*)
**Birth weight (grams)**	1883.39 ± 900		2014.8 ± 930.9		(t = -2.17, *P* = .03*)
**1-min Apgar scores, (Mean ± SD),Median (Min–Max)**	(6 ± 1.4),6 (2–8)		(6 ± 1.4),6 (1–8)		(U = 109,265.5, *P* = .02*)
**5-min Apgar scores, (Mean ± SD), Median (Min–Max)**	(8 ± 1.1),9 (4–10)		(8 ± 1.3),9 (1–10)		(U = 108,682, *P* = .008*)
**10-min Apgar scores, (Mean ± SD), Median (Min–Max)**	(10 ± .6), 10(6–10)		(10 ± .63), 10(4–10)		(U = 115,146, *P* = .08)
**IVH, n (%)**	106	28.3	114	17.9	(X2 = 7.74, *P* = .005*)
**PDA, n (%)**	140	37.3	182	28.6	(X2 = 5.47, *P* = .02*)
**Bleeding tendency, n (%)**	183	48.8	129	20.3	(X2 = 88.1, *P* < .001*)
**Pulmonary Hemorrhage, n (%)**	65	17.3	9	1.4	(X2 = 89.9, *P* < .001*)
**CVS stability at admission**	276	73.6	427	67.1	**(X2 = .02, *P* = .08)**
**Bronchopulmonary dysplasia (BPD)**	31	8.3	28	4.4	**(**^**MC**^*P* = .017*)

**X**^**2**^; Pearson Chi-Square test

^*^Significant (*p* < 0.05)

*t* Independent t test, *IUGR* intrauterine growth retardation

U Mann Whitney test, *CVS instability* cardiovascular instability, *CS* C section, *NVD* normal vaginal delivery, *BPD* bronchopulmonary dysplasia, *IVH* intraventricular hemorrhage, *PDA* patent ductus arteriosus

As the identifying the cause of thrombocytopenia is mostly provisional and is not confirmed. We identified first and second possible cause of thrombocytopenia according to the clinical situation and positive laboratory investigations at the time of thrombocytopenia. The current study identified the etiologies of thrombocytopenia (first possible cause), with 28% attributed to placental insufficiency, 24% to sepsis, 14.9% to perinatal hypoxia, 9.1% to alloimmune, 7.5% to double volume exchanges, 3.7% to isoimmune, 3.7% to post-therapeutic hypothermia, 3.2% to congenital anomalies, 3.2% to NEC, 1.1% to consumptive coagulopathy, and 0.5% due to neonatal anemia causes, S-Table 2.

Table 2 represents patterns of thrombocytopenia in relation to the etiology. In Table 2, thrombocytopenic patients were classified according to severity into mild, moderate and severe. Perinatal asphyxia was the most common cause of mild and severe thrombocytopenia, while klebsiella induced sepsis was the most common cause of moderate thrombocytopenia. In addition, Table 2 showed onset of thrombocytopenia in studied patients. 69% of thrombocytopenic patients showed early onset thrombocytopenia (EOT) and 31% showed late onset thrombocytopenia (LOT). The LOT is further divided into LOT, very late onset thrombocytopenia (VLOT), and extremely late onset thrombocytopenia (ELOT). Also, Table 2 assessed patients regarding onset offset, duration and nadir of thrombocytopenia.

**Table 2:**
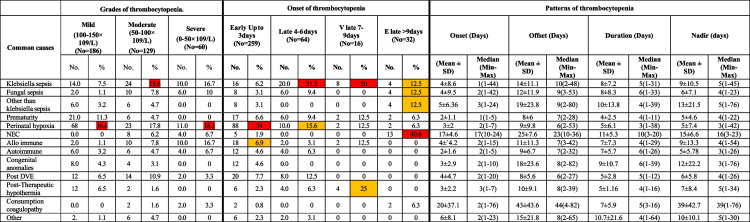
Distribution of common causes according to the grades of thrombocytopenia (mild, moderate, severe), onset of thrombocytopenia (early, late, very late, and extremely late) and the patterns of thrombocytopenia (onset, offset, duration and Nadir)

^#^The response was not mutually exclusive

The red color indicates the most prevalent cause in each type of thrombocytopenia

The orange color represents the second prevalent cause in each type of thrombocytopenia

*DVE* double volume exchange, *NEC* necrotizing enterocolitis, *V late* very late, *E late* extremely late

Regarding maternal risk factors, our univariate analysis in s-table-3 showed that pregnancy induced hypertension (PIH), premature rupture of membranes (PROM), systemic lupus erythromatosis (SLE), idiopathic thrombocytopenic purpura (ITP) & anti-phospholipid syndrome, gestational diabetes mellitus (GDM), perinatal hypoxia, maternal thrombocytopenia were risk factors for the development of thrombocytopenia. Multivariate logistic analysis of maternal and neonatal risk factors for thrombocytopenic patients (Table 3) showed that sepsis early onset sepsis (EOS) and late onset sepsis (LOS), and NEC were highly statistically significant in predictor of thrombocytopenia and they were constant in the three constructed models. Maternal immune diseases and gestational diabetes as risk factors of thrombocytopenia were significant only in model2 and model3, respectively.

**Table 3:** Multivariate analysis of maternal and neonatal risk factors for thrombocytopenic patients

	**Unstandardized Coefficients**		**Sig**	**Odd Ratio**	**95% Confidence Interval for Odd Ratio**	
	**B**	**Std. Error**			**Lower Bound**	**Upper** **Bound**
**Model 1**						
• **Constant**	-1.15	.14	.001*	-	-	-
• **NEC (Yes)**	.811	.291	.005*	2.250	1.272	3.981
• **Perinatal hypoxia (Yes)**	.048	.144	.737	1.049	.792	1.391
• **IUGR(Yes)**	.420	.251	.095	1.521	.929	2.490
• **Preterm (< 37 weeks)**	.157	.149	.293	1.170	.873	1.567
• **Sepsis**			< .001*			
o **Early**	1.576	.203	< .001*	4.833	3.249	7.190
o **Late**	1.882	.242	< .001*	6.567	4.083	10.562
Dependent variable; presence of thrombocytopenia Reference group; No NEC, No Perinatal hypoxia, No IUGR, > 37 weeks and No-sepsis						
**Model 2**						
• **Constant**	-1.2	.21	.001*	-	-	-
• **NEC (Yes)**	.856	.343	.013*	2.355	1.202	4.611
• **Perinatal hypoxia (Yes)**	.223	.209	.285	1.250	.830	1.882
• **Preterm (< 37 weeks)**	-.006	.211	.978	.994	.658	1.503
• **IUGR(Yes)**	.373	.321	.246	1.451	.774	2.723
• **Sepsis**			.001*			
o **Early**	1.376	.305	.001*	3.960	2.177	7.203
o **Late**	1.436	.446	.001*	4.202	1.754	10.068
• **Maternal immune disease (Yes)**	.985	.367	.007*	2.678	1.306	5.494
Dependent variable; presence of thrombocytopenia Reference group; No-NEC, No-Perinatal hypoxia, No-IUGR, > 37 weeks, no-sepsis and no-immune disease						
**Model 3**						
• **Constant**	-1.2	.17		-	-	-
• **PIH(Yes)**	-.334	.844	.692	.716	.137	3.741
• **GDM(Yes)**	1.523	.563	.007*	4.587	1.521	13.84
• **NEC (Yes)**	.701	.310	.024*	2.016	1.098	3.701
• **Perinatal hypoxia (Yes)**	.136	.164	.407	1.145	.831	1.578
• **Preterm (< 37 weeks)**	.191	.170	.260	1.210	.868	1.688
• **IUGR(Yes)**	-.169	.357	.636	.845	.420	1.699
• **Sepsis**			< .001*			
o **Early**	1.267	.241	< .001*	3.549	2.212	5.693
o **Late**	1.497	.304	< .001*	4.466	2.461	8.107

Dependent variable; presence of thrombocytopenia

Reference group; No-PIH, No-DM, No-NEC, No-Perinatal hypoxia, No-IUGR, > 37 weeks and No-sepsis

*PIH* pregnancy induced hypertension, *PROM* premature rupture of membranes, *GDM* gestational diabetes mellitus, *IUGR* intrauterine growth retardation, *NEC* necrotizing enterocolitis

Maternal immune thrombocytopenia includes systemic lupus erythromatosis (SLE), idiopathic thrombocytopenia ( ITP) and anti-phospholipid syndrome

The mortality rate among thrombocytopenic patients was 43.2%. Table 4 shows multivariate logistic analysis of mortality for thrombocytopenic patients. Pulmonary hemorrhage, intraventricular hemorrhage (IVH) and cardiovascular instability were risk factors for mortality in thrombocytopenic patients with Odds ratio (IQR) 20.9(6.743–64.9), 5.7(2.1–15.8) and 4 (1.9–8.3), respectively.

**Table 4:** Multivariate logistic analysis of risk factors for mortality in thrombocytopenic patients

**Model**	**Unstandardized Coefficients**		**Sig**	**Odd Ratio**	**95% Confidence Interval for Odd Ratio**	
	**B**	**S.E**				
					**Lower**	**Upper**
• Constant	-2.294	.330	.001*			
• Klebsiella sepsis (Positive)	.512	.541	.343	1.669	.578	4.817
• Fungal sepsis (Positive)	.039	.808	.961	1.040	.213	5.065
• Other than Klebsiella (Positive)	1.197	.689	.083	3.309	.857	12.777
• Pulmonary Hemorrhage (Positive)	3.041	.578	.001*	20.927	6.743	64.943
• Preterm (Yes)	.449	.442	.310	1.566	.658	3.726
• LBW(Yes)	.618	.413	.135	1.855	.825	4.171
• Invasive ventilation (Yes)	.280	.485	.564	1.323	.511	3.422
• CVS instability (Yes)	1.752	.515	.001*	5.767	2.101	15.828
• Severe IVH((Yes)	1.386	.375	.001*	4.000	1.918	8.345
• Mild thrombocytopenia			.654			
• Moderate thrombocytopenia	-.251	.347	.471	.778	.394	1.538
• Severe thrombocytopenia	.148	.465	.750	1.160	.466	2.884

Dependent variable; not survive

Refence group; negative Klebsiella sepsis, negative Fungal sepsis, negative Other than Klebsiella sepsis, non-Pulmonary Hemorrhage, non-Preterm, non-LBW, non-Invasive ventilation, non-CVS instability, non IVH and mild severity of thrombocytopenia)

S-Table 4 and s-Table 5 show laboratory investigations in both thrombocytopenic and nonthrombocytopenic patients. ALT, AST, PT, creatinine, direct bilirubin, lymphocytic count, CRP were significantly higher and Hb was significantly lower in thrombocytopenic group. S-Table 6 and s-Fig. 1 show management options in the thrombocytopenic patients, and S-Table 7 and S-Fig. 2 demonstrate antibiotic taken at time of thrombocytopenia.

## Discussion

AUMH is a tertiary center that receives mothers with several risk factors from 4 governments. Most of patients admitted to NICU are premature infants. They represent 69.3% of thrombocytopenic patients and 62.3% of non-thrombocytopenic patients. The prevalence of thrombocytopenia as revealed by most of studies is 18- 35% [10–14].While, thrombocytopenia was seen in 37.1% of patients in NICU of AUMH according to the current work.

In our study, the non-thrombocytopenic groups had a significantly higher birth weight than the thrombocytopenic groups (2014.8 ± 930.9 vs 1883.39 ± 900 g). According to Saini et al.'s cross-sectional observational investigation, low birth weight (68.06%) was the main risk factor for infant thrombocytopenia in their hospital [15]. The bulk of analyzed data also revealed that the incidence of thrombocytopenia in neonates admitted to NICUs was negatively correlated with the infants' birth weights and gestational ages, with a frequency of about 70% in the extremely low birth weight (ELBW, ≤ 999 g) [14].

Sepsis was the most common cause of thrombocytopenia in the current study. Positive blood cultures, lymphocytic count, CRP were significantly higher in thrombocytopenic group. This can be explained by higher prevalence of sepsis in the thrombocytopenic group. The etiological profile of newborn with thrombocytopenia was similarly documented by Nandyal et al., with neonatal infections (22.2%) and prematurity (38.3%) being the most prevalent causes [16]. Bleeding, pulmonary hemorrhage and IVH were significantly higher in thrombocytopenic than nonthrombocytopenic group. A relationship between platelet count and bleeding has not been established. In a prospective, multicenter study conducted by Stanworthe et al., it was found that although one-third of the neonates enrolled in the study developed thrombocytopenia of < 20 × 10^9^ platelets/L, 91% of them did not experience major hemorrhage [17].

According to von Lindern et al., the severity of thrombocytopenia is unrelated to IVH or death, despite the fact that thrombocytopenic newborns constitute a high-risk category (more unstable and sicker than non-thrombocytopenic neonates) [18]. In 33% of thrombocytopenic neonates with IVH grade 2 or higher, IVH occurred before the occurrence of thrombocytopenia. Their data confirmed that the risk of IVH in neonates was a complex mechanism related to a wide variety of factors, of which low platelet counts was only one. In our study, 69% of patients had early onset thrombocytopenia (in the first 3 days) and IVH developed in the first 3 days of life in 28% of patients. In addition, IVH occurrence was significantly higher in thrombocytopenic group. Duppre et al., found that cellular and humoral coagulation disorders play a more significant role in the occurrence of IVH in neonates than thrombocytopenia [19].

According to the current research patent ductus arteriosus (PDA), bronchopulmonary dysplasia (BPD) and retinopathy of prematurity (ROP), were more common in thrombocytopenic than non-thrombocytopenic group. Boo et al., conducted a prospective study to determine the predictors of failed PDA closure after the administration of a single course of indomethacin. They hypothesized that poor thrombus formation in the ductal lumen may be caused by low platelet count, which they found to be an independent risk factor for failure of closure [20].

Yan et al., argued that platelets may participate development of pulmonary microvascularization in premature infants with BPD [21]. May be due to platelet activation and thrombopoietin (TPO) regulation. They also suggested that in infants with BPD, platelet consumption was at least partly responsible for the low peripheral platelet count. Four percent of our thrombocytopenic patients had ROP, with 1.9% occurring on the right side and 2.1% on the left side. Several studies investigated the association between thrombocytopenia and ROP. Vinekar et al., described a case of aggressive posterior ROP with severe thrombocytopenia that resolved after serum platelet transfusions [22].

Stratifying the etiological profile of thrombocytopenia against grades of thrombocytopenia, timing of onset, nadir, duration and off-set can be beneficial for the clinician. There was a higher percent of EOT in our study than LOT. Nandyal et al. reported that EOT was present in 43.4% of neonates, while the remaining 56.5% of neonates presented with LOS [16]. Jeremiah et al. observed EOT in 84.84%, and Eslami Z et al. observed EOT in 75.3% [23, 24]. Our study, however, went beyond simply classifying neonatal thrombocytopenia as EOT or LOT and added a further classification to the LOT with 17.3% occurring between days four and six (LOT), 4.3% between days seven and nine (vey LOT), and 8.3% after nine days of life (extremely LOT). Table 2 categorized the principal reasons for neonatal thrombocytopenia according to the timing of onset.

During NICU admissions, sixteen percent of the thrombocytopenic individuals in the current research had severe thrombocytopenia. On the other hand, Nandyal et al. reported severe thrombocytopenia in 65.6% of neonates [16]. The higher prevalence of thrombocytopenia in their research may be attributed to the study's setting in a referral center, as well as the higher proportion of neonatal prematurity and septicemia.

Our study displayed the distribution of common causes of thrombocytopenia according to severity, as depicted in (Table 2). Sepsis, particularly from Klebsiella and fungal infections, often leads to moderate to severe thrombocytopenia. However, thrombocytopenia resulting from infections other than Klebsiella tended to be less severe degree. NEC, alloimmune thrombocytopenia, autoimmune thrombocytopenia, and consumption coagulopathy were usually presented with more severe thrombocytopenia.

Prematurity, congenital anomalies, and thrombocytopenia resulting from therapeutic hypothermia generally contributed to mild and moderate degrees of thrombocytopenia. Perinatal hypoxia caused thrombocytopenia across both ends of the spectrum, affecting both mild and severe degrees.

In our work, we identified different patterns of thrombocytopenia in relation to the different etiologies of neonatal thrombocytopenia, Table-2. Patterns illustrate the distribution of causes against onset, duration, timing of nadir, and the offset of thrombocytopenia. It can be more helpful to investigate the etiology of neonatal thrombocytopenia based on the time of onset alone.

Our results aligned with some studies summarized as follows: [25, 26] typical patients with EOT had either a low normal platelets count at birth (150–200 × 10^9^/L) or borderline thrombocytopenia (100–150 × 10^9^/L). Their platelets counts then decreased slowly to a nadir on days 4–5 of life before recovering to (> 150 × 10^9^/L) by 7–14 days. In contrast, patients with LOT had a different pattern. It progressed rapidly, with a platelet nadir reached within 24–48 h. Thrombocytopenia was typically severe (< 50 × 10^9^/L) and prolonged. A study by Von-Lindern et al. showed that thrombocytopenia was detected at a mean of 2 days after birth (range 0–56 days). The mean duration of thrombocytopenia was 9 days (range 0–112 days) [18]. The duration of thrombocytopenia in the mild, moderate, severe, and very severe groups was 5, 8, 10, and 16 days, respectively.

In the current study we studied etiology-oriented different patterns of thrombocytopenia, hopefully, this might help the clinician in earlier and easier identification of etiology, more understanding pathophysiological mechanisms behind the illness of the neonates and rapid management of the condition. Furthermore, the current research might encourage different units to develop individualized approach of thrombocytopenia based on their etiology- oriented patterns.

Table 2 can help the clinician through identification of the pattern of thrombocytopenia in prediction of nadir (lowest and most critical point) and offset, and severity of thrombocytopenia. The prediction of offset, nadir and severity patterns can help clinicians to stop and avoid unnecessary lines of management.

A high mortality rate was reported among thrombocytopenic patients. Thrombocytopenic patients were more critically ill than non-thrombocytopenic patients. Positive blood cultures ALT, AST, PT, creatinine, direct bilirubin, lymphocytic count, CRP were significantly higher and Hb was significantly lower in thrombocytopenic group. Mortality incidence according to the severity of thrombocytopenia displayed the following distribution: severe cases (61.7%), moderate cases (41.8%), and mild cases (38.2%), as depicted in S-Table-1. The multivariate logistic analysis of risk factors for mortality in thrombocytopenic patients revealed that pulmonary hemorrhage, cardiovascular instability, and severe IVH were highly statistically significant in predicting mortality (as shown in Table 4).

Many limitations can be acknowledged in this cross-sectional study. Firstly, the presence of a non-electronic medical record system to collect data, and its retrospective nature. Secondly, the inability to follow the long-term neurological outcome of survived thrombocytopenic patients. Thirdly, failing to come to agreement upon a cut point of where platelets transfusion could be applied in management of thrombocytopenia. Fourthly, the patterns of thrombocytopenia can be applied to low and middle income countries rather than high income countries.

## Conclusion

Early onset neonatal thrombocytopenia is commonly related to perinatal hypoxia and prematurity. Late and very-late onset neonatal thrombocytopenia is commonly sepsis related to klebsiella while, extremely late thrombocytopenia is related to NEC.

Mild thrombocytopenia is related to hypoxia and prematurity. Moderate thrombocytopenia is commonly caused by klebsiella infection followed by perinatal hypoxia. Severe thrombocytopenia can happen with alloimmune thrombocytopenia and perinatal asphyxia. Therefore, identification of patterns of thrombocytopenia can help the clinician to suspect the correct etiology of thrombocytopenia.

Sepsis, NEC, maternal diabetes and maternal immune diseases are the most important perinatal predictors of thrombocytopenia. Pulmonary hemorrhage, IVH and Cardiovascular instability are the most important predictors of mortality in thrombocytopenic patients.

## Supplementary Information

Supplementary Material 1.

## Data Availability

The datasets generated during and/or analyzed during the current study are available from the corresponding author on reasonable request.

## Abbreviations

IVH: Intraventricular hemorrhage
IUGR: Intrauterine growth retardation
BPD: Bronchopulmonary dysplasia
NEC: Necrotizing enterocolitis
ROP: Retinopathy of prematurity
EOS: Early onset sepsis
LOS: Late onset sepsis
EOT: Early onset thrombocytopenia
LOT: Late onset thrombocytopenia
PDA: Patent ductus arteriosus
PIH: Pregnancy induced hypertension
PROM: Premature rupture of membranes
SLE: Systemic lupus erythromatosis
ITP: Idiopathic thrombocytopenic purpura
GDM: Gestational diabetes mellitus

## References

[1] Abebe Gebreselassie H, Getachew H, Tadesse A, Mammo TN, Kiflu W, Temesgen F, Dejene B. Incidence and Risk Factors of Thrombocytopenia in Neonates Admitted with Surgical Disorders to Neonatal Intensive Care Unit of Tikur Anbessa Specialized Hospital: A One-Year Observational Prospective Cohort Study from a Low-Income Country. J Blood Med. 2021;30(12):691–7. 10.2147/JBM.S321757 PMC8335549 34366682

[2] Ulusoy E, Tüfekçi Ö, Duman N, et al. Thrombocytopenia in neonates: causes and outcomes. Ann Hematol. 2013;92:961–7. 23519382 10.1007/s00277-013-1726-0

[3] Josefsson EC, Vainchenker W, James C. Regulation of Platelet Production and Life Span: Role of Bcl-xL and Potential Implications for Human Platelet Diseases. Int J Mol Sci. 2020;14;21(20):7591.1 10.3390/ijms21207591 PMC7589436 33066573

[4] Sillers L, Van Slambrouck C, Lapping-Carr G. Neonatal Thrombocytopenia: Etiology and Diagnosis. Pediatr Ann. 2015;44(7):e175–80. 26171707 10.3928/00904481-20150710-11 PMC6107300

[5] Gunnink SF, Vlug R, Fijnvandraat K, van der Bom JG, Stanworth SJ, Lopriore E. Neonatal thrombocytopenia: etiology, management and outcome. Expert Rev Hematol. 2014;7(3):387–95. 24665958 10.1586/17474086.2014.902301

[6] Baer VL, Lambert DK, Henry E, Christensen RD. Severe thrombocytopenia in the NICU. Pediatrics. 2009;124(6):e1095–100. 19917581 10.1542/peds.2009-0582

[7] Resch E, Hinkas O, Urlesberger B, Resch B. Neonatal thrombocytopenia-causes and outcomes following platelet transfusions. Eur J Pediatr. 2018;177(7):1045–52. 29705932 10.1007/s00431-018-3153-7 PMC5997104

[8] Shah S, Khan W. Prevalence of Thrombocytopenia in Neonates Admitted at Rehman Medical Institute Peshawar. J Wazir Muhammad Inst Paramed Technol (JWMIPT). 2021;1(2):22–5.

[9] Zekry S, Hamed E, Hassanen F, Abdel-Aziz S. Incidence and Risk Factors for Neonatal Thrombocytopenia among Newborns admitted to NICU of Assiut University Children’s Hospital-A Prospective Observational Study. Ann Neonatol J. 2022;4(1):7–26.

[10] Carr R, Kelly AM, Williamson LM. Neonatal thrombocytopenia and platelet transfusion - A UK perspective. Neonatology. 2015;107(1):1–7. 25301082 10.1159/000365163

[11] Sparger K, Deschmann E, Sola-Visner M. Platelet Transfusions in the Neonatal Intensive Care Unit. Clin Perinatol. 2015;42(3):613–23. 26250921 10.1016/j.clp.2015.04.009 PMC4535179

[12] Sparger KA, Assmann SF, Granger S, Winston A, Christensen RD, Widness JA, et al. Platelet transfusion practices among very-lowbirth-weight infants. JAMA Pediatr. 2016;170(7):687–94.5. 27213618 10.1001/jamapediatrics.2016.0507 PMC6377279

[13] Iqbal Q, Bashir C, Mushtaq S, Ahmad A, Baba AR. Thrombocytopenia and other hematological parameters in culture positive neonatal sepsis and their impact. J Pediatr Infect Dis. 2013;8(1):25–9.

[14] Bolat F, Kiliç SÇ, Oflaz MB, Gülhan E, Kaya A, Güven AS, et al. The prevalence and outcomes of thrombocytopenia in a neonatal intensive care unit: A three-year report. Pediatr Hematol Oncol. 2012;29(8):710–20. 23013425 10.3109/08880018.2012.725454

[15] Saini R, Saini P, Sehra R, Saini L, Gehlot Y. Thrombocytopenia burden and its associating risk factors: A cross-sectional study at a tertiary care set up. Int Multispec J Health. 2017;3:237–43.

[16] Nandyal SS, Shashikala P, Sahgal V. Study of thrombocytopenia in neonat al intensive care unit. Indian J Patho Oncol. 2016;3(l):55–9.

[17] Stanworth SJ, Clarke P, Watts T, Ballard S, Choo L, Morris T, et al. Prospective, observational study of outcomes in neonates with severe thrombocytopenia. Pediatrics. 2009;124(5):e826–34. 19841111 10.1542/peds.2009-0332

[18] Von Lindern JS, van den Bruele T, Lopriore E, Walther FJ. Thrombocytopenia in neonates and the risk of intraventricular hemorrhage: a retrospective cohort study. BMC Pediatr. 2011;11:16. 21314921 10.1186/1471-2431-11-16 PMC3045959

[19] Duppré P, Sauer H, Giannopoulou EZ, Gortner L, Nunold H, Wagenpfeil S, et al. Cellular and humoral coagulation profiles and occurrence of IVH in VLBW and ELWB infants. Early Hum Dev. 2015;91(12):695–700. 26529174 10.1016/j.earlhumdev.2015.09.008

[20] Boo NY, Mohd-Amin I, Bilkis AA, Yong-Junina F. Predictors of failed closure of patent ductus arteriosus with indomethacin. Singapore Med J. 2006;47(9):763–8. 16924357

[21] Yan L, Ren Z, Wang J, Xia X, Yang L, Miao J, et al. The Correlation Between Bronchopulmonary Dysplasia and Platelet Metabolism in Preterm Infants. Front Pediatr. 2021;9:670469. 34900853 10.3389/fped.2021.670469 PMC8652141

[22] Vinekar A, Hegde K, Gilbert C, Braganza S, Pradeep M, Shetty R, et al. Do platelets have a role in the pathogenesis of aggressive posterior retinopathy of prematurity? Retina. 2010;30(4 Suppl):S20–3. 20224477 10.1097/IAE.0b013e3181cafc30

[23] Jeremiah ZA, Oburu JE. Pattern and prevalence of neonatal thrombocytopenia in Port Hartcourt. Nigeria Pathol Lab Med Intern. 2010;2:27–31.

[24] Eslami Z, Lookzadeh MH, Noorishadkam M, Hashemi A, Ghilian R, Pirdehghan A. Thrombocytopenia and Associated Factors in Neonates Admitted to NICU during Years 2010_2011. Iran J Pediatr Hematol Oncol. 2013;3(1):205–15. PMC3915438 24575265

[25] McPherson RJ, Juul S. Patterns of thrombocytosis and thrombocytopenia in hospitalized neonates. J Perinatol. 2005;25(3):166–72. 15578031 10.1038/sj.jp.7211230

[26] Christensen RD, Baer VL, Henry E, Snow GL, Butler A, Sola-Visner MC. Thrombocytopenia in Small-for-Gestational-Age Infants. Pediatrics. 2015;136(2):e361–70. 26216323 10.1542/peds.2014-4182 PMC4906543

