# Supplementary material for: Prevalence and determinants of thrombocytopenia in newborn unit at Alexandria University Hospital: a three-year report including 1000 patients
Source: BMC Pediatr. 2024 Dec 10;24:805. doi: 10.1186/s12887-024-05170-7 (PMC11629486; doi:10.1186/s12887-024-05170-7)
Supplement: Supplementary file 1 — Supplementary Material 1. [file 12887_2024_5170_MOESM1_ESM.docx]

**S-Table** **1: Relation of degree of severity thrombocytopenia with some parameters**

|  | **Severe**  **(0-50× 10^9^/L)**  **(No=60)** | **Moderate**  **(50-100× 10^9^/L)**  **(No=129)** | **Mild**  **(100-150× 10^9^/L)**  **(No=186)** | **Test of significance**  **(p)** |
| --- | --- | --- | --- | --- |
| **Gestational age (weeks) (Mean ± SD), (Min-Max)** | (32.9±4.1),(25-40) | (33.9±3.8),(28-39) | (34±4),(26-41) | (F=1.6, P=.2) |
| **Birth weight (g) median (Min-Max)** | 1245(500–3500) | 1740 (650–4000) | 1790 (500–4450) | (H=2.7, P=.25) |
| **1-minute Apgar scores** | 4±1.5 | 6±1.1 | 8±1.6 | (F=215, P<.001*) |
| **5-minute Apgar scores** | 5±1.4 | 7±1.3 | 9±1.6 | (F=146, P<.001*) |
| **10-minute Apgar scores** | 9±1.4 | 9±1.6 | 9±1.6 | (F=2.6, P=.07) |
| **Bleeding tendency, n (%)** | 8(13.3%) | 29(22.5%) | 33(17.7%) | **(^MC^P=.79)** |
| **Pulmonary Hemorrhage, n (%)** | 4(6.7%) | 13(10.1%) | 11(5.9%) | **(^MC^P=.67)** |
| **IUGR, n (%)** | 18(30%) | 14(10.8%) | 7(3.8%) | **(X^2^=33.5, P<.001*)** |
| **Sepsis, n (%)** | 34 (56.7) | 54(41.8) | 68(36.6) | **(X^2^=7.5, P=.02*)** |
| **IVH, n (%)** | 45 (75%) | 50(38.8 %) | 37(19.9%) | **(X^2^=61.5, P<.001*)** |
| **PDA, n (%)** | 39(65%) | 46(35.7 %) | 33(17.7%) | **(X^2^=48.4, P<.001*)** |
| **Mortality, n (%)** | 37(61.7) | 54(41.8) | 71(38.2) | **(X^2^=10.34, P=.005*)** |

F;One Way ANOVA test

H;Kruskal Wallis test

Mcp;MontCarlo test

*; statistically significant

**S-Table 2: Possible causes of thrombocytopenia**

| 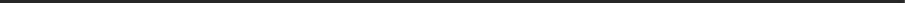  **1^st^ possible causes** | **No.** | **%** |
| --- | --- | --- |
| - Placental insufficiency | 106 | 28.3 |
| - Sepsis | 92 | 24.5 |
| - Early onset sepsis | 55 | 14.7 |
| - Late onset sepsis | 37 | 9.8 |
| - Perinatal hypoxia | 56 | 14.9 |
| - Allo- immune thrombocytopenia | 34 | 9.1 |
| - Post double volume blood exchange to cases of severe jaundice | 28 | 7.5 |
| - Auto- immune thrombocytopenia | 14 | 3.7 |
| - Post therapeutic hypothermia | 14 | 3.7 |
| - Congenital anomalies | 12 | 3.2 |
| - Necrotizing enter colitis (NEC) | 12 | 3.2 |
| - Consumption coagulopathy | 4 | 1.1 |
| - Neonatal anemia | 2 | 0.5 |
| - Incidental (unexplained) | 2 | 0.5 |
| **2^nd^ possible causes** | 70 | 18.7 |
| - Placental insufficiency |  |  |
| - Perinatal hypoxia | 30 | 8 |
| - Sepsis | 40 | 10.6 |
| - Early onset sepsis | 26 | 6.9 |
| - Late onset sepsis | 14 | 3.7 |
| - NEC | 16 | 4.3 |
| - Consumption coagulopathy other than DIC | 10 | 2.7 |
| - Post double volume exchange of severe neonatal jaundice | 8 | 2.1 |
| - Alloimmune thrombocytopenia | 8 | 2.1 |
| - Disseminated intra vascular coagulopathy (DIC) | 8 | 2.1 |
| - Inborn error of metabolism | 6 | 1.6 |
| - Unexplained | 4 | 1.1 |
| - Autoimmune thrombocytopenia | 2 | 0.5 |
| - Post therapeutic hypothermia | 2 | 0.5 |
| - TORCH | 2 | 0.5 |
| - Genetic congenital malformation | 2 | 0.5 |
| - Polycythemia | 2 | 0.5 |
| **Other possible causes** | 58 | 15.5 |
| - Placental insufficiency |  |  |
| - Sepsis | 36 | 9.6 |
| - Early onset sepsis | 21 | 5.6 |
| - Late onset sepsis | 15 | 4 |
| - Perinatal hypoxia | 26 | 6.9 |
| - Alloimmune thrombocytopenia | 8 | 2.1 |
| - Inborn error of metabolism | 6 | 1.6 |
| - Consumption coagulopathy | 6 | 1.6 |
| - Drug related as indomethacin | 6 | 1.6 |
| - Autoimmune thrombocytopenia | 4 | 1.1 |
| - NEC | 4 | 1.1 |
| - Congenital malformations | 4 | 1.1 |
| - Unexplained | 2 | 0.5 |

**S-Table 3:** **Univariate analysis of maternal and neonatal risk factors for thrombocytopenic patients**

|  | **Study groups** | | | |  |
| --- | --- | --- | --- | --- | --- |
|  | **Thrombocytopenic patients**  **(No.=375)** | | **Non-Thrombocytopenic patients**  **(No.==636)** | | **Test of significance**  **(p)** |
|  | **No.** | **%** | **No.** | **%** |  |
| **Maternal risk factors** |  |  |  |  |  |
| - PIH | 78 | 20.8 | 78 | 12.3 | **(X^2^=19.9, P<.001*)** |
| - PROM | 26 | 6.93 | 36 | 5.66 | **(X^2^=4.2, P=.04*)** |
| - SlE & ITP & antiphosplipid | 6 | 1.60 | 2 | 0.31 | **(X^2^=7.35, P=.007*)** |
| - GDM | 24 | 6.40 | 9 | 1.42 | **(X^2^=25, P=.001*)** |
| - Perinatal hypoxia | 155 | 41.33 | 287 | 45.13 | **(X^2^=6.6, P=.01*)** |
| - Maternal thrombocytopenia | 19 | 5.07 | 14 | 2.20 | **(X^2^=4.39, P=.04*)** |
| **Neonatal risk factors** |  |  |  |  |  |
| - IUGR | 37 | 9.87 | 39 | 6.13 | **(X^2^=4.7, P=.03*)** |
| - Preterm (<37 weeks) | 260 | 69.33 | 396 | 62.26 | **(X^2^=5.2, P=.024*)** |
| - NEC | 35 | 9.33 | 25 | 3.93 | **(X^2^=12.2, P<.001*)** |
| - Sepsis |  |  |  |  | **(X^2^=106, P<.001*)** |
| - - Early | 85 | 22.7 | 46 | 7.23 |  |
| - - Late | 71 | 18.7 | 27 | 4.24 |  |

Abbreviations :PIH pregnancy induced hypertension, PROM premature rupture of membranes, SLE systemic lupus erythromatosis, ITP idiopathic thrombocytopenia, GDM gestational diabetes mellitus, IUGR intrauterine growth retardation. NEC necrotizing enterocolitis.

**S-Table 4: CBC investigations among thrombocytopenic patients at time of admission Vs at the start thrombocytopenia**

| **CBC investigations** | **At time of admission**  **Mean ±SD (Min-Max)** | | **At the start thrombocytopenia**  **Mean ±SD (Min-Max)** | | **Test of significance**  **(p)** |
| --- | --- | --- | --- | --- | --- |
| - Hemoglobin (g/dl) | 15.2±3.5 | 4.3-24 | 13.9±3.8 | 4.4-24 | (t=8.5,P<.001*) |
| - Hct (%) | 42.8±10.75 | 4.2-70 | 39.83±10.3 | 12.6-68 | (t=6.1,P<.001*) |
| - MCV(fl) | 107.3±10.5 | 76-129.6 | 101.7±13.2 | 68.9-136.5 | (t=9.8,,P<.001*) |
| - MCH(pg) | 37.8±5.1 | 26-52.3 | 35.5±5.6 | 26-52.3 | (t=8.3,P<.001*) |
| - MCHC(fl) | 37.7±5 | 26-52 | 35.5±5.6 | 26-52 | (t=8.4,P<.001*) |
| - White blood counts (Wbcs) (10^3^/ul) | 14.84±11.6 | 1.9-79 | 15.47±11.7 | .4-70 | (t= -1.05,P=.3) |
| - Neutrophils (10^3^/ul) | 7.15±6.7 | .2-43 | 8.11±7.9 | .2-45 | (t= -2.6, P=.01*) |
| - Lymphocytes (10^3^/ul) | 6.2±6.78 | 1.1-47.7 | 5.8±6.4 | .12-36 | (t=1.5,P=.12) |
| - Mean platelet volume(fl) | 8.8±2.6 | 4.7-33.4 | 8.84±2.1 | 4.6-13.5 | (t=-.73,P=.5) |
| - Reticulocytes (%) | 6.36±8.9 | .2-55 | 4.5±2.9 | .2-11.2 | (t=1.9, P=.06) |

t; Paired t test *;statistically significant

**S-Table 5: Comparing between Thrombocytopenic patients Vs Non thrombocytopenic patients as regards some laboratory investigations**

|  | **Thrombocytopenic**  **patients**  **(No.=375)** | **Non-Thrombocytopenic patients**  **(No.=636)** | **Test of significance**  **(p)** |
| --- | --- | --- | --- |
| **CBC investigations** | | | |
| - Hemoglobin (g/dl) | 15.2±3.5 | 15.5±2.5 | (t=-1.5,P=.13) |
| - Hct (%) | 42.8±10.75 | 46.3±28.5 | (t=-2.3,P=.018*) |
| - MCV(fl) | 106.9±12.5 | 104.1±10.9 | (t=3.7,P<.001*) |
| - MCH(pg) | 37.94±6.6 | 36.4±4.2 | (t=4.3,P<.001*) |
| - MCHC(fl) | 34.25±4.6 | 34.8±2.5 | (t=-2.6,P=.009*) |
| - Wbcs (10^3^/ul) | 14.84±11.6 | 14.4±10.3 | (t=.5,P=.6) |
| - Neutrophils (10^3^/ul) | 7.15±6.7 | 7.6±6.5 | (t=-1.2,P=.23) |
| - Lymphocytes (10^3^/ul) | 6.2±6.78 | 5.2±4.8 | (t=2.7,P=.006*) |
| - Platelet (10^3^/ul) | 176.4±105.3 | 263.8±94.7 | (t= -13,P<.001*) |
| - Mean platelte volume(fl) | 8.8±2.6 | 8.7±1.96 | (t=.8,P=.4) |
| - Reticulocytes(%) | 6.36±8.9 | 4.5±5.8 | (t=1.7,P=.09) |
| **Kidney &Liver functions &Electrolytes &Blood gases** | | | |
| - BUN (mg/dl) | 22.9±17.1 | 21.7±11.9 | (t= -.05, P=.9) |
| - Urea (mg/dl) | 45.5±31.8 | 46.2±29.1 | (t= -.37, P=.7) |
| - Creatinine (mg/dl) | .95±.7 | .8±.5 | (t=3.5, P<.001*) |
| - ALT (U/L) | 25.2±31.5 | 20.4±19.4 | (t= 2.9, P=.003*) |
| - AST (U/L) | 85.3±120.3 | 63.4±84.6 | (t=3.5, P<.001*) |
| - Albumin (g/dl)   Median(Min-Max) | 2.8(.2-14) | 3(.2-14) | (U= 111080,P=.12) |
| - Na (mmol/l) | 137.6±10.9 | 138.4±11.3 | (t= -.3, P= .7) |
| - K (mmol/l) | 4.7±.97 | 4.8±.9 | (t= -1.6, P= .1) |
| - Ca (mg/dl) | 8.7±3.4 | 8.4±1.3 | (t= -.08, P= .9) |
| - Phosphors (mg/dl) | 5.6±2.1 | 5.8±1.3 | (t= -.28,P=.7) |
| - Cl (mmol/l) | 104.6±7.1 | 104.6±7.5 | (t= -.08, P=.9) |
| - Alkaline phosphatase(U/L) | 73.7±87.7 | 76.6±80.8 | (t= -.35, P=.7) |
| - TSB (mg/dl)   Median(Min-Max) | 5.9(.4-44) | 5.7(.2-44) | (U= 115552,P=.67) |
| - DSB (mg/dl) | .6±1.3 | .5±.7 | (t=2.14, P=.03*) |
| - ABG PH | 7.3±.11 | 7.33±.12 | (t= -.34, P=.7) |
| - Pco2 (mmHg) | 39.9±12.88 | 39.7±13.4 | (t=.214,P=.8) |
| - Po2 (mmHg) | 73.5±51 | 73.9±47.1 | (t=-.122, P=.9) |
| - Lactate mmol/l   Median(Min-Max) | 2(.9-22) | 1.9(.9-38.1) | (U= 1737.5, P=.7) |
| - CRP (mg/l) | 22.9±54.2 | 10.8±25.9 | (t= 4.8, P<.001*) |

ALT: Alanine Transaminase. AST: Aspartate Amino Transferase .TSB: Total Serum Bilirubin. DSB: Direct Serum Bilirubin. ABG: Arterial Blood Gases. CRP: C Reactive Protein .

**s-Table 5: Comparing between Thrombocytopenic patients Vs Non thrombocytopenic patients as regards some laboratory investigations (continue)**

|  | **Thrombocytopenic**  **patients**  **(No.=375)** | | **Non-Thrombocytopenic patients**  **(No.=636)** | | **Test of significance**  **(p)** |
| --- | --- | --- | --- | --- | --- |
| **Bleeding test** | | | | | |
| - APTT sec | 52.9±26 | | 51.4±22.9 | | (t=.85, P=.4) |
| - PT Activity % | 54.9±22.5 | | 57.03±21.9 | | (t=-1.8, P= .06) |
| - PT time sec | 18.5±15.9 | | 15.67±7.14 | | (t=3.5, P<.001*) |
| - INR | 1.57±.9 | | 1.54±1.6 | | (t= .32,P=.75) |
| **CSF analysis ^#^** | | | | | |
| - Neutrophils cell/ul | 0(0-1750) | | 0(0-1750) | | (U= 2.3, P=.3) |
| - Lymphocytes cell/ul | 0(0-6000) | | 0(0-6000) | | (U= 1.2, P=.6) |
| - Red blood cells (RBCS) cell/ul | 10(0-8000) | | 0(0-8000) | | (U= 4.5,P=.1) |
| - Protein mg/dl | 71(0-418) | | 71.5(0-418) | | (U= .37, P=.8) |
| - Glucose mg/dl | 60(0-305) | | 65(2-305) | | (U= 2.2, P=.4) |
| **Blood culture** | **No.** | **%** | **No.** | **%** |  |
| - Klebsila | 55 | 14.7 | 60 | 9.4 | **(X^2^=.17, P=.9)** |
| - Candida | 9 | 2.4 | 11 | 1.7 |  |
| - Ecoli | 5 | 1.3 | 6 | 0.9 |  |
| - Actinobacter | 6 | 1.6 | 7 | 1.1 |  |
| - Enterococcus | 7 | 1.9 | 7 | 1.1 |  |
| - MRSA | 7 | 1.9 | 8 | 1.3 |  |
| - Pseudomonas | 3 | 0.8 | 4 | 0.6 |  |

#; Data described by Median (Min-Max) . U; Mann Whitney test

PT:prothrobin time, APTT: activated partial thromboplastin, INR:international normalized ratio.MRSA: Methicillin Resistant Staphylococcus Aureus.

**S-Table 6: Management of thrombocytopenic patients as a first option:**

|  | **No.** | **%** |
| --- | --- | --- |
| **Upgrade antibiotics** | 180 | 48.0 |
| **observation and CBC follow up with no intervention and only bleeding survey** | 168 | 44.8 |
| **Sepsis work up including triple tap** | 100 | 26.7 |
| **Platelet transfusions** | 80 | 21.3 |
| **Plasma transfusion** | 28 | 7.5 |
| **IVIG** | 10 | 2.7 |
| **Steroids** | 10 | 2.7 |

**S-Figure 1:** Management of thrombocytopenic cases.

# S-Table 7: Antibiotics use at time of management of thrombocytopenia

|  | **No.** | **%** |
| --- | --- | --- |
| - Amikin | 161 | 42.9 |
| - Claforan | 127 | 33.9 |
| - Meronam | 126 | 33.6 |
| - Vancomycin | 119 | 31.7 |
| - Tienam | 107 | 28.5 |
| - Diflucan | 91 | 24.3 |
| - Sulprazone | 84 | 22.4 |
| - Levofloxicin | 60 | 16.0 |
| - Colstin | 35 | 9.3 |
| - Gentamycin | 20 | 5.3 |
| - Tygacil | 17 | 4.5 |
| - Unasyin | 15 | 4.0 |
| - Fungizone | 10 | 2.7 |
| - Flagyl | 6 | 1.6 |
| - Cipro | 5 | 1.3 |
| - Linzeolid | 5 | 1.3 |
| - Dalacin | 5 | 1.3 |
| - Fosfomycin | 3 | 0.8 |
| - Fortum | 3 | 0.8 |
| - V fend | 2 | 0.5 |

#The response was not mutually exclusive

**%**


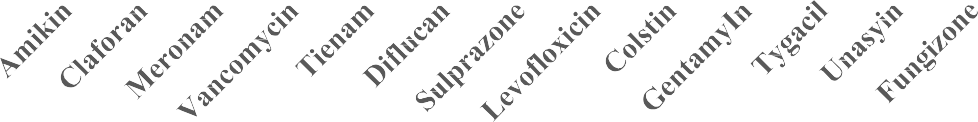


**2.7**

**4**

**4.5**

**5.3**

**9.3**

**16**

**24.3 22.4**

**28.5**

**31.7**

**33.9 33.6**

**42.9**

**50**

**45**

**40**

**35**

**30**

**25**

**20**

**15**

**10**

**5**

**0**

**S-Figure 2:** Antibiotics use at time of management of thrombocytopenia
